# Surgery in COVID-19 patients: operational directives

**DOI:** 10.1186/s13017-020-00307-2

**Published:** 2020-04-07

**Authors:** Federico Coccolini, Gennaro Perrone, Massimo Chiarugi, Francesco Di Marzo, Luca Ansaloni, Ildo Scandroglio, Pierluigi Marini, Mauro Zago, Paolo De Paolis, Francesco Forfori, Ferdinando Agresta, Alessandro Puzziello, Domenico D’Ugo, Elena Bignami, Valentina Bellini, Pietro Vitali, Flavia Petrini, Barbara Pifferi, Francesco Corradi, Antonio Tarasconi, Vittoria Pattonieri, Elena Bonati, Luigi Tritapepe, Vanni Agnoletti, Davide Corbella, Massimo Sartelli, Fausto Catena

**Affiliations:** 1 Emergency Surgery Unit & Trauma Center, Pisa University Hospital, Pisa, Italy; 2 Emergency and Trauma Surgery, Maggiore Hospital, Parma, Italy; 3 General Surgery Dept., Sansepolcro Hospital, Sansepolcro, Italy; 4 General, Emergency and Trauma Surgery Dept., Bufalini Hospital, Cesena, Italy; 5 General Surgery Dept., Busto Arsizio Hospital, Busto Arsizio, Italy; 6 General Surgery Dept., Ospedaliera San Camillo Forlanini, Rome, Italy; 7 General and Emergency Surgery Dept., A. Manzoni Hospital, Lecco, Italy; 8 General Surgery Dept., Ospedale Gradenigo, Torino, Italy; 9 ICU Dept., Pisa University Hospital, Pisa, Italy; 10 General Surgery Dept., Ospedale Civile, Adria, Italy; 11 General Surgery Dept., Salerno University Hospital, Salerno, Italy; 12 General Surgery Dept., Policlinico Gemelli University Hospital, Rome, Italy; 13 ICU Dept., Parma University Hospital, Parma, Italy; 14 Igiene and Public Health Dept., Parma University Hospital, Parma, Italy; 15 ICU Dept., Chieti University Hospital, Chieti, Italy; 16 ICU Dept., San Camillo Forlanini Hospital, Rome, Italy; 17 ICU Dept., Bufalini Hospital, Cesena, Italy; 18 Neuro ICU Dept., Papa Giovanni XXIII Hospital, Bergamo, Italy; 19 General and Emergency Surgery, Macerata Hospital, Macerata, Italy; 20 General, Emergency and Trauma Surgery, Pisa University Hospital, Via Paradisia 1, 56100 Pisa, Italy

**Keywords:** Coronavirus, COVID-19, Epidemic, Pandemic, Mass casualties, Management, Resources, Criticalities, WSES

## Abstract

The current COVID-19 pandemic underlines the importance of a mindful utilization of financial and human resources. Preserving resources and manpower is paramount in healthcare. It is important to ensure the ability of surgeons and specialized professionals to function through the pandemic. A conscious effort should be made to minimize infection in this sector. A high mortality rate within this group would be detrimental.

This manuscript is the result of a collaboration between the major Italian surgical and anesthesiologic societies: ACOI, SIC, SICUT, SICO, SICG, SIFIPAC, SICE, and SIAARTI. We aim to describe recommended clinical pathways for COVID-19-positive patients requiring acute non-deferrable surgical care. All hospitals should organize dedicated protocols and workforce training as part of the effort to face the current pandemic.

## Background

The current COVID-19 pandemic, “when the destructive effects of natural or man-made forces overwhelm the ability of a given area or community to meet the demand for health care” [1], demands the best disaster/mass casualty incident (MCI) response. During MCIs, preserving financial and human resources is crucial. A good organization and a preventive approach are mandatory in the phase of MCI response called mitigation. In order to minimize resource exhaustion, the use of surgical appliances and staff must be well pondered and balanced [2]. Surgeons and sub-specialized workers in general are a valuable resource during MCI. Infection or death of sub-specialized staff must be minimized to preserve the ability to face surgical emergencies and associated activities that will continue to occur or perhaps increase during MCI. In fact, any lack of specialized teams occurring during a pandemic cannot be easily addressed by reintegrating retirees or replenishing the ranks with new staff, which would also be inevitably associated with a lowered standard of care, hence, the requirement to skeletonize surgical activities during a pandemic. When possible, all surgical procedures on all suspected COVID-19 patient should be postponed until confirmed infection clearance. Minimal staff should be involved when deferral is not possible. If a large number of senior surgeons is exposed to infected patients, the possibility for them to become infected and require self-isolation is real and could potentially result in a dangerous shortage of senior expertise within surgical teams. Resource usage should be carefully considered when planning scheduled procedures, particularly with regard to materials, staff, devices, intensive care beds, blood components, etc. Caring for resource-intensive patients might be controversial during MCIs.

This manuscript is the result of a collaboration between the major Italian surgical and anesthesiologic societies: ACOI, SIC, SICUT, SICO, SICG, SIFIPAC, SICE, and SIAARTI. We aim to describe recommended clinical pathways for COVID-19-positive patients requiring acute non-deferrable surgical care.

## Main text

All known or suspected COVID-19-positive patients requiring surgical intervention must be treated as positive until proven otherwise in order to minimize infection spread. Protocolized clearly defined pathways must be available to healthcare professionals caring for these patients. Allocating dedicated senior staff to key management roles is crucial to minimize COVID-19 spread. All staff must be specifically trained to don, doff, and dispose of personal protection equipment (PPE) including masks (level 2 or 3 filtering face piece (FFP) depending on the aerosol-generating risk level), eye protection, double non-sterile gloves, gowns, suites, caps, and socks (Table 1).

**Table 1:** Necessary personal protection equipment

Personal protection equipment	
FFP2 facial mask	
FFP3 facial mask (in case of maneuvers at high risk of generating aerosolized particles)	
Disposable long sleeve waterproof coats, gowns, or Tyvek suits	
Disposable double pair of nitrile gloves	
Protective goggles or visors	
Disposable head caps	
Disposable long shoe covers	
Alcoholic hand hygiene solution	

*FFP* filtering face piece

**Fig. 1:**
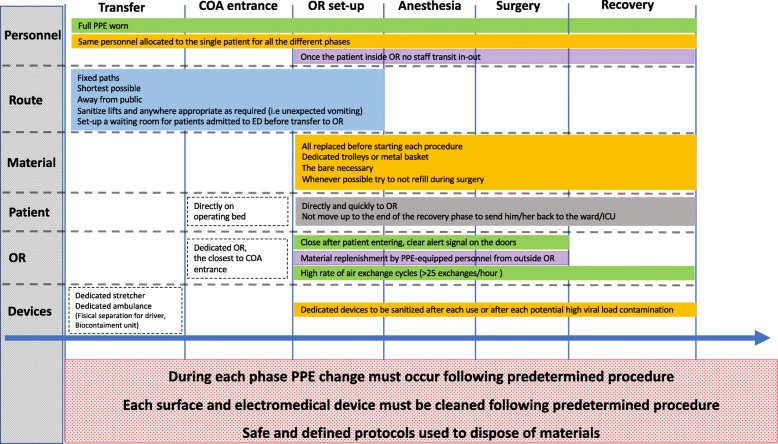
COVID-19 surgical patients management flowchart

In-transit surgical patients proceeding through the theater block must not stop in the anesthetic bay, recovery room, or any place other than the COVID-dedicated operating room (OR). They must be taken directly to a designated OR that must be adequately marked with clearly visible door signs. In the event that the scheduled surgical procedure does not require a general anesthetic and if the clinical situation allows, patients should continue to wear a protective mask for the entire duration of the procedure (Fig. 1).

It is important to underline how all non-COVID patients must be protected. Established separate pathways must exist to keep suspected/infected patients apart from non-COVID ones. PPEs or at least masks must be enforced for all non-COVID patients during all in-hospital transfers in order to minimize infection risk in the event that they cross the path or come in proximity of a COVID patient. Careful planning and segregation of infected patients may help minimize staff shortages related to uncontrolled viral spread.

### Location

Designated COVID operating areas (COA) must be allocated to COVID patients’ urgent/emergent operating. The OR closest to the entrance of the theater block entrance should be the first one designated to COVID patients. When multiple procedures must be simultaneously performed, operating rooms must be utilized in order of proximity to the theater block entrance in order to minimize environmental contamination in the theater block.

### Patient transport

Patient transit to and from the COA must be as quick as possible. A pre-defined direct path must be kept as short as possible and away from other patients and people in general within the hospital in order to minimize the chances of infection. If inter-hospital patient transfer or transfer from other buildings within the hospital is required, a dedicated vehicle should be used. Transfer personnel should be specifically trained and equipped with PPEs. The patient’s compartment in the transport vehicle is ideally kept separate from the driver. A Biocontainment unit may be utilized. If a patient is taken to the COA from any adjacent premise, a stretcher might be used for transport. All precautionary measures apply to the use of the stretcher and to the personnel responsible for the transfer (Table 1, Fig. 1) both during and after transport is completed, with immediate sanitization required (Tables 2 and 3). Utilized lifts must be sanitized. If any unexpected contamination occurs during transport (i.e., patient vomiting or else), adequate dedicated sanitization should take place. A dedicated specifically trained 24/7 cleaning team from the local contracted cleaning service might prove a valuable resource.

**Table 2:** Sanitization sequence

Surface and electromedical sanitization sequence	
1. Clean with chloro-derivate solution	
2. Rinse and dry	
3. Disinfect with chloro-derivate solution in a concentration **≥** 0.1% or 1000 ppm; time of contact must be superior to 1 min	

*ppm* parts per million

**Table 3:** COVID-19 surgical patients’ management

Key aspects in COVID-19 surgical patient management	
All suspected or infected patients must be managed with the maximum attention.	
All personnel in contact with the patient must wear PPE.	
Transfers must be protected.	
Infected patients must be moved as little as possible through the hospital.	
Transfer routes must be precisely planned and be as short as possible.	
The COVID operating area should be in a dedicated and possibly separate area.	
COVID operating room must be dedicated and as close as possible to the entrance of the theater block.	
Disposable material should be preferred.	
Minimal material should be used for each intervention.	
Transport personnel should be the same from transport origin to destination.	
Once the patient has entered, the OR doors must be closed.	
Operators (i.e., surgeon, anesthetist, nurses, technicians) should enter the OR in a timely manner to minimize exposure to infected patients.	
Personnel involved in the intervention should not leave the OR during the procedure.	
High OR air exchange cycles are recommended (> 25 exchanges/h).	
Clinical documentation must remain outside the OR	
At the end of each intervention all disposable materials must be disposed of and all surfaces and electromedical devices accurately cleaned and disinfected.	
PPE must be removed and disposed of outside the OR in dedicated doffing areas ensuring the virus is not transmitted to the healthcare worker.	
OR and surrounding donning/doffing areas must be sanitized as soon as possible after each procedure.	
After each procedure, all involved personnel, whenever possible, should shower.	
Recovery phase after surgery must be done in OR, before transfer the ward/ICU.	

Any non-intubated patient must wear a surgical mask, disposable waterproof gloves, disposable cap, and shoe covers during transport. When possible, the patient’s hands should be sanitized before transport. Transport operators must sanitize hands and wear PPEs before transfer and should minimize contact with patients. Coded routes should be followed and hospital public areas avoided. Anyone crossing the path of an infected patient should be preemptively alerted in order to minimize contact. Well-organized logistics will contribute to minimizing disposables wastage. Dedicated well-identifiable containers for infectious-risk health waste (IRHW) should be used for potentially infected disposables. Lastly, COVID patients should be transported in the most professional and confidential way possible in order to minimize unjustified alarmism. Dedicated areas allocated to infected patients awaiting transfer to the COA must be preemptively identified in the emergency department. The patient’s transfer from the emergency department to the COA should be streamlined in order to avoid all unnecessary contacts. Each hospital should provide a step by step, well-defined path pre-allocating some corridors and elevators to COVID patients.

## COVID operating area

It is important to minimize the total number of operators working in the designated COA. Whenever possible, it is important to minimize to number of people working on a single infected case; ideally, this should also apply to cases spanning over multiple shifts. Operations for COVID patients might be organized with a dedicated on-call shift. This might require overnight or out of hours activities to optimize resource usage. This approach might facilitate segregation between COVID and non-COVID patients, who will continue to require surgical care. PPEs and stock required for hand hygiene must be constantly replenished within the COA. A specifically allocated filter area designed for COVID patients to enter the COA must be equipped with PPEs, hand hygiene station, and a dedicated IRHW bins. Handling of potentially infected linen should be adequately managed too. The use of machinery intended to facilitate moving and transferring patients should be minimized. All COA doors must be kept closed (including accessory rooms, sterilization spaces), and any equipment not necessary for the intervention must be moved away from COVID patients transit route.

### Taking charge of the patient in COVID operating area

Special attention should be given to what, in non-COVID times, is routine practice. Staff taking responsibility for positive or suspected infected patients must be limited to those who need to be primarily involved in each operation. A record must be kept of all operators involved in procedures on potentially infected patients. Personnel equipped with full PPEs must receive the patient in the COA, transfer the patient to the operating room minimizing environmental contamination and, after time-out, proceed to move the patient on the operating table in the allocated OR. All non-intubated patients must wear a surgical mask. Medical records must remain outside the OR and must be consulted and updated there after adequate doffing. Intraoperative document consultation is discouraged and should be minimized.

### Operating room preparation

Negative pressure ORs would be ideal to minimize infection risk [3, 4]. However, ORs are normally designed to have positive pressure air circulation. A high air exchange cycle rate (≥ 25 cycles/h) contributes to effectively reduce the viral load within ORs [2]. Equipment kept in each OR must be minimized to what is strictly necessary on a case to case basis. Once the operation starts, all efforts must be made to use what is available in the room and minimize staff transiting in and out the OR, in order to minimize infection risk. Standard anesthetic trolleys should be replaced with dedicated pre-prepared ones with minimal but adequate stock. All required surgical material (i.e., stitches, scalpel blades) must be preemptively prepared in a sterilizable steel wire basket. Dedicated IRHW containers must be used for infected and sharp disposable instruments. Alcoholic solution for hand hygiene must always be available. Avoiding non-strictly necessary commonly used non-disposable devices is recommended. Disposable material in general should be preferred, including linen. All operators (i.e., surgeon, anesthetist, nurses, technicians) should enter the OR timely, aiming to minimize time spent within the OR itself. Once in the OR, they should not leave until the operation is completed, and once out they should not re-enter.

### Personnel dressing

All operators must wear the required PPE before meeting the infected patient. The patient’s receiving personnel inside the COA filter area must perform hand hygiene and wear full PPE.

While taking care of infected patients, gloves should be changed immediately after contact with infected material (objects, surfaces, etc.) or if any damage occurs. Operator with a beard should exert special attention to the fit of the mask ensuring adequate protection.

Some procedures likely to generate aerosolized particles have been associated with increased coronavirus transmission: tracheal intubation, non-invasive ventilation, tracheostomy, cardiopulmonary resuscitation, and manual ventilation before intubation and bronchoscopy [5, 6]. An FFP3 mask should be therefore worn by operators working closer to the patient during these procedures.

Given the conjunctiva’s susceptibility to viral transmission, it is important to wear visors or goggles to protect the eyes from potential exposure of viral particles [7].

### Anesthesiologic consideration

Careful anesthesiologic planning is recommended to minimize any infection potentially associated with unexpected complex endotracheal intubation procedures. A more liberal use of intubation might be justified in patients with acute respiratory failure, bypassing non-invasive ventilation techniques (e.g., CPAP or biPAP) in order to minimize the transmission risks [5]. Disposable airway equipment should be preferred. Medical and nursing staff must be equipped with FFP3 filters during laryngoscopy and intubation [5]. Intubations techniques with the highest chance of first-time success should be preferred to avoid repeated airway instrumentation [4, 5]. Awake intubation techniques should be avoided. At the end of these procedures, all staff directly performing the procedure must immediately replace the first pair of gloves and other PPEs in case heavy contamination risk exists (i.e., in the event that vomiting, coughing, or else has occurred). Fiberscope intubation, unless specifically indicated, should be avoided as it may generate aerosolization [5]. Rapid sequence intubation (RSI) should be considered to avoid manual ventilation and potential aerosolization. If manual ventilation is required, small current volumes should be used. If available, a closed suction system should be preferred during airway aspiration. Disposable covers should be used whenever possible to reduce equipment contamination. If a patient is transferred directly from the intensive care unit, a dedicated transport ventilator should be utilized. In order to reduce aerosolization risks, the gas flow should be turned off and the endotracheal tube clamped with forceps when switching from the portable device to the OR ventilator [4]. When possible, a dedicated ventilator should be used in the OR for general anesthesia in positive or suspected positive COVID-19 patients. Invasive procedures like for example the placement of intercostal catheters, central venous catheters, or similar should be performed at the patient’s bedside, rather than in the OR. When a general anesthetic is required, a HEPA (high-efficiency particulate air) filter should be connected to the patient end of the breathing circuit and another one between the expiratory limb and the anesthetic machine [2, 6]. Alternatively, for pediatric patients or other patients in whom additional dead space or the weight of the filter may be problematic, the HEPA filter must be placed at the expiratory end of the circuit (before the exhalation re-enters the ventilator). The gas sampling tube must also be protected by a HEPA filter. Both HEPA filters and soda lime must be changed after each case [4]. At the end of the surgery, during the recovery phase, the patient must be assisted directly in the OR until ready to be transferred back to the inpatients place of stay. The time patients spend returning to wards must be reduced in order to minimize contact between COVID-positive patients and the surrounding environment.

### Intraoperative management

The OR door must be kept closed at all times and clear signs should discourage unnecessarily entering the room. Supplying materials to the OR during surgery should also be discouraged. The scout nurse, in collaboration with the operating surgeon, should anticipate what is needed during the operation before the same starts. Surgeons should preferably perform the operation with what is available in the OR once the operation started. Any essential retrieval of necessary equipment should be done by staff outside the OR. Personnel present in the OR during surgery must not leave the room. Electromedical devices (i.e., ultrasound) and surfaces must be used with adequate protective cover and adequately sanitized at the end of the operation. The surgical team will drape the patient according to the surgical procedure, replacing the surgical mask with FFP2 filter and wearing long shoe covers before doing so. All personnel in direct contact with the patient must wear a double pair of gloves at all times, even while operating. After the patient left the OR, logistics should allow as much time as possible before the next procedure takes place, to reduce possible air contamination. This time depends on the number of air exchanges/hour of the specific room. Air exchange cycles should be increased whenever possible to ≥ 25 exchanges/h [2]. After the case, all areas at risk of contamination must be cleaned and disinfected (Table 2). Efforts should be made to minimize the contamination risk associated with specimens sent to the pathology department. No data currently exist on COVID-19 viral load in bodily fluids or tissue samples.

### PPE undressing/removal

Staff not directly involved in the patient’s care should leave the OR at the end of the operation and remove all PPEs in a dedicated doffing area following the sequence described below. A clean area should be accessed only after the doffing procedure is complete. All used PPEs must be disposed of through IRHW containers. Scrubs must be replaced after each procedure following showering, whenever possible. Personnel responsible for transferring the patient away from the operating room must follow separate access routes and wear PPEs different from the ones worn in the OR.

### Instructions for PPE removal

The healthcare professional must take all care not to become infected while removing PPE; this must be done through an adequate procedure preventing re-contamination of the operator's clothing and hands. The first pair of gloves is likely to be heavily contaminated and must be removed first. All other PPEs must be considered infected as well and removed with care during the doffing procedure, especially if the patient had a cough. Protective suite, shoe cover, and head cap must be subsequently removed. Face mask and glasses must be then removed, taking care to handle the face mask by the ear laces and without touching its external side. The second pair of gloves must be removed as the very last PPE and hands disinfection with hydro-alcoholic solution must be accurately performed immediately after.

### Environmental sanitization

The OR and surrounding exchange areas must be sanitized as soon as possible after each procedure, with particular attention to all objects used when caring for infected patients. Similarly, all areas where COVID patients have transited must be carefully sanitized too. All personnel must contribute to maintain a clean environment including floors and surfaces in general. All potentially infected single-use materials should be disposed of through IRHW containers. Reusable materials should be decontaminated, washed, dried, and or disinfected/sterilized, based on the likelihood of infection. Electromedical equipment (i.e., ventilator, radiological equipment) must be cleaned with chloro-derivate solution, rinsed and dried, and then disinfected with chloro-derivate solution in a concentration **≥** 0.1% or 1000 ppm (parts per million) with contact time superior to 1 min [8, 9] (Table 2). Full PPE must be worn during the sanitizing procedure. Disposable materials only (i.e., double gloves, paper towel) should be used for cleaning. Anything disposable kept inside the OR during the operation must be disposed of through IRHW containers, even if not used.

### Waste disposal

It is advisable to set up a dedicated container for hazardous medical waste immediately outside the OR, to immediately dispose of all contaminated disposable material and PPEs. Containers should be closed and sealed before being transferred to the collection point. All sharps should be disposed of in a dedicated rigid plastic container. PPE should be worn when closing and transporting containers and removed immediately after. Any visibly damaged or contaminated container must be promptly replaced.

### Linen management

Linen can be contaminated and must therefore be handled and transported with care, aiming to prevent infection spread. Disposable laundry should be preferred, when possible. All linen (sheets, pillowcases, crossbars, etc.) should be handled wearing PPE during collection, not placed on surfaces or floors, but directly inside dedicated containers. These must be sealed and immediately sent for cleaning and sterilization, limiting them being left outside the OR.

## Conclusion

Instituting precise well-established plans to perform undeferrable surgical procedures and emergencies on COVID-19-positive patient is mandatory. Hospitals must prepare specific internal protocols and arrange adequate training of the involved personnel.

## Data Availability

Not applicable

## Abbreviations

MCIs: Mass casualty events
PPE: Personal protection equipment
FFP: Filtering face piece
COA: COVID operating area
IRHW: Infectious-risk health waste
HEPA: High-efficiency particulate air
RSI: Rapid sequence intubation
